# iTrust—A Trustworthy and Efficient Mapping Scheme in Elliptic Curve Cryptography

**DOI:** 10.3390/s20236841

**Published:** 2020-11-30

**Authors:** Hisham Almajed, Ahmad Almogren, Mohammed Alabdulkareem

**Affiliations:** Department of Computer Science, College of Computer and Information Sciences, King Saud University, Riyadh 11633, Saudi Arabia; 438105079@student.ksu.edu.sa (H.A.); kareem@KSU.EDU.SA (M.A.)

**Keywords:** concatenation method, elliptic curve cryptography, encoding phase, mapping phase, mobile crowed-sourcing systems, probability method

## Abstract

Recently, many platforms have outsourced tasks to numerous smartphone devices known as Mobile Crowd-sourcing System (MCS). The data is collected and transferred to the platform for further analysis and processing. These data needs to maintain confidentiality while moving from smartphones to the platform. Moreover, the limitations of computation resources in smartphones need to be addressed to balance the confidentiality of the data and the capabilities of the devices. For this reason, elliptic curve cryptography (ECC) is accepted, widespread, and suitable for use in limited resources environments such as smartphone devices. ECC reduces energy consumption and maximizes devices’ efficiency by using small crypto keys with the same strength of the required cryptography of other cryptosystems. Thus, ECC is the preferred approach for many environments, including the MCS, Internet of Things (IoT) and wireless sensor networks (WSNs). Many implementations of ECC increase the process of encryption and/or increase the space overhead by, for instance, incorrectly mapping points to EC with extra padding bits. Moreover, the wrong mapping method used in ECC results in increasing the computation efforts. This study provides comprehensive details about the mapping techniques used in the ECC mapping phase, and presents performance results about widely used elliptic curves. In addition, it suggests an optimal enhanced mapping method and size of padding bit to secure communications that guarantee the successful mapping of points to EC and reduce the size of padding bits.

## 1. Introduction

**Figure 1:**
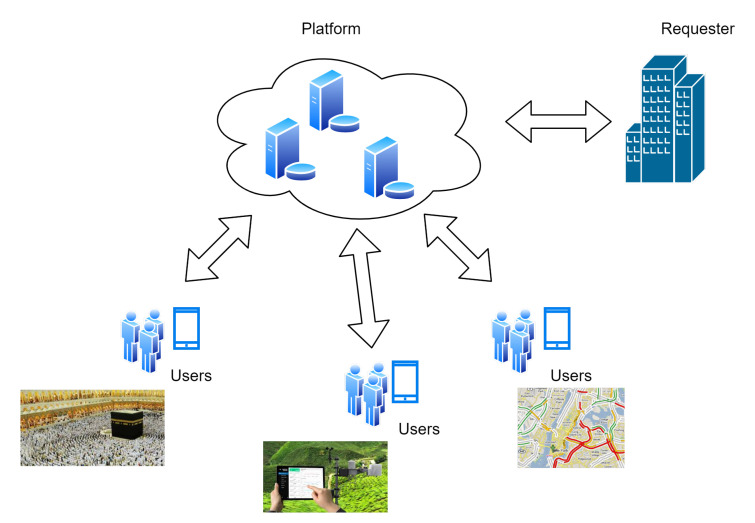
Applications of a Mobile Crowd-sourcing System.

The rapid increase of smartphones that are equipped with many useful sensors [1,2], such as, Global Positioning System (GPS), gyroscopes, meter sensors, etc., and their ability to connect to the Internet using 3G/4G/5G connectivity, have increased the growth and the use of MCSs [3]. MCS is an approach to outsource tasks to several smartphones to collect specific data into a centralized platform to analyze and process these data in order to share it with specific users. There are many applications behind MCSs, such as monitoring the environment, monitoring the road traffic, etc., as depicted in Figure 1. For example, Alhogail, Areej, et al [4] developed an Umrah Electronic Guide system that facilitated GPS Positioning and counting techniques to self-guide Pilgrims during Umrah activities. However, MCSs transmitted these data over an insecure network (the Internet). Therefore, the confidentiality of these data and the identity of the smartphone users need to be maintained. In addition, the MCS uses low capability devices (i.e., smartphones) to collect and transmit these data; thus, the cryptography system needs to address these limitations.

Lightweight encryption schemes such as elliptic curve cryptography (ECC) are becoming increasingly desirable due to the growing interest surrounding the use of low computing power devices, particularly those associated with the Internet of Things (IoT) and wireless sensor networks (WSNs) [5,6,7,8,9,10,11,12]. Encryption schemes of this kind satisfy the need to maintain the confidentiality and integrity of transmitted data without compromising performance. Schemes such as ECC consist of many phases [13,14]. These phases are: initialising, encoding, mapping, encryption, signing, verifying, decrypting, decoding, and —finally—converting to the message [15]. Given the multi-phase nature of ECC and several other encryption schemes, it is possible that security flaws, vulnerabilities, and performance overheads may increase [16]. For this reason, deriving value from the use of ECC depends on the effective implementation of each phase, as well as robust and reliable performance evaluation. Therefore, this study helps to address MCS and the expansion of cities and urbanization for disseminating the services through efficient ECC and an enhanced mapping phase.

The mapping phase in ECC consists of a mathematical equation that represents the elliptic curve, which is given as follows:

(1) $$y^{2}\equiv x^{3}+a\;x+b\;mod\;p$$

where $a,b\in Z_{p}$ and $4a^{3}+27b^{2}\neq 0\;mod\;p$. A given *x* is said to be mapped to the elliptic curve if and only if there exists a corresponding *y* that satisfies Equation (1). If no such *y* exists, then *x* is not mapped to the elliptic curve. The crucial advantage associated with mapping points to an elliptic curve stems from an exploitation of the elliptic curve discrete logarithm problem (ECDLP) [17], which constitutes the base of ECC. However, if a message *M*, encrypted using ECC, did not map to an elliptic curve (i.e., the *x* value of *M* has no corresponding *y*), then it is necessary to increment *x* and recalculate until *y* is found [18,19,20]. However, the increment to *x* changes *M*, thereby resulting in the wrong decoding phase for the retrieval of *M*. Thus, to secure messages in the encoding and decoding phases, certain bits should be concatenated to mapping points to avoid changing the original message. In the decoding phase, these padding bits are removed from the mapped points in a safe and secure manner.

### Preliminary: Elliptic Curve Cryptography (ECC)

ECC is used widely in constrained environments, particularly those relying on low computing power devices (e.g., IoT and WSNs) [21,22,23]. It provides the same level of cryptographic hardness as do other asymmetric cryptography protocols, but it is marked by small key sizes and higher performance [24,25]. For instance, cryptography schemes relying on a 1024-bit key with the Rivest-Shamir-Adleman (RSA) algorithm achieve the same level of cryptographic hardness that is associated with ECC with a 160-bit key. The result of the difference in key sizes leads to the low capabilities devices performing more effective computing [26,27]. The base of hardness in ECC is the discrete logarithm structure of elliptic curves over finite fields [28,29], where the ECC is used to exchange keys, and to encrypt transmitted message between two parties [30,31,32]. Additionally, ECC is used to ensure the integrity of transmitted messages and non-repudiation using elliptic curve digital signature algorithms (ECDSA) [33,34]. Many schemes use ECC to secure communications, and these schemes vary depending on the type of ECC that used [35,36,37]. For instance, some schemes use ECC to exchange a shared key between two parties, other schemes are applied to secure the confidentiality and integrity of messages.

Two operations are defined on the elliptic curve. The first is addition ‘+’. Let *P* and *Q* be two points on the elliptic curve, where $P=(x_{1},y_{1})$ and $Q=(x_{2},y_{2})$. Then the operation P+Q on the elliptic curve is defined as $=\left(x_{1},y_{1}\right)+\left(x_{2},y_{2}\right)=\left(x_{3},y_{3}\right)$. Notably, $\left(x_{3},y_{3}\right)$ is the third point on the elliptic curve that intersects with the line between *P* and *Q*. If P=Q, then $P+P=\left(x_{1},y_{1}\right)+\left(x_{1},y_{1}\right)=2P$ is defined as point doubling.

The major operation in ECC is group multiplication [38,39]. It is the number of operations of group point doubling. It consists of two variables: firstly, *d*, which is an integer known only to the participants, and which serves as the private key; and secondly, $G=(x_{i},y_{i})$, which is the base point on the elliptic curve. The public key is the product of the operation dG, which is the *d* doubling times for the base point *G*. This operation results in point $\left(x_{j},y_{j}\right)$. ECC’s security stems from the computational hardness associated with finding *d* when the adversary has the base point *G* and the public key [17]. The abovementioned ECDLP stipulates that there is no efficient algorithm that yields *d* in polynomial time.

To secure communications, maintain data integrity, and exchange keys, ECC consists of several phases [13,14]. Certain ECC applications use these phases to provide authenticated encryption (AE), while others use the phases to offer integrity or confidentiality. The following are the phases involved in ECC:

- Initialising and generating system parameters, which includes defining the elliptic curve and base points, and calculating the private key $P_{r}$ and the public key $P_{u}$.
- Encoding the plaintext message *M* to numerical values for use in the next phase.
- Mapping the numerical values to the elliptic curve to exploit the ECDLP.
- Encrypting the mapped values.
- Hashing the encrypted message (i.e., for signing).
- Verifying the received ciphertext.
- Decrypting the ciphertext.
- Decoding the decrypted ciphertext to convert it into numerical values.
- Converting numerical values into the plaintext message *M*.

In ECC, the initializing and generating phase requires the greatest effort in terms of computation. This is because it is during this stage that the computation of the keys is completed. As previously noted, the public key is obtained by calculating the d×G where the *d* is the private key known by the sender only and *G* is the EC base point [40,41]. Several strategies are available for optimising this phase, for instance, improving scalar multiplication on the elliptic curve. The encoding phase involves techniques that convert the message characters into numerical values. This is necessary because the ECC cryptosystem deals with numbers [42]. Similarly, the mapping phase facilitates the mapping of the numerical values outputted from the previous phase to the elliptic curve, where its equation is used to identify the elliptic curve’s pair points [43]. Next, the encryption phase involves it being represented by the summation of the mapped points and the public key. Finally, the transmitted ciphertext is signed to authenticate the sender and, in this way, secure it against tampering. The remaining phases invert the preceding phases, in which the recipient verifies the received ciphertext, decrypts the ciphertext, decodes the numerical values, and converts it into plaintext.

This paper’s objective is to present an effective mapping phase performance in ECC. To achieve this objective, several secondary goals must be addressed. In particular, it is necessary to gain insight into the current techniques used to pad bits in the mapping phase. Additionally, for each technique, this paper presents a performance analysis and evaluation. In turn, this paper provides a comprehensive investigation of the mapping phase for several known and widely used elliptic curves. For instance, secp192k1, NIST-224, and secp256k1 are examined [44,45,46]. Finally, this paper proposes effective padding bit values and a method that guarantees successful mapping, as well as efficiency using the least number of bits. A performance evaluation of the proposed method is given, comparing its results against those of the schemes presented in the related work section. The rest of this paper is organised as follows: the next section discusses related work on the mapping phase; the following section presents the study of padding bits and the effective bit values for padding encoded messages; the subsequent section describes the evaluation performance of the proposed method; and the last section offers concluding remarks, as well as avenues for future work.

## 2. Related Works

Existing schemes use ECC to reduce the encryption processing overhead. This is valuable due to the limitations of low computing power devices [47,48,49]. However, many of these schemes provide scant details about how a message should be padded in order to map it successfully to the elliptic curve [50,51,52]. Noteworthily, existing schemes have introduced significant enhancements in many areas of the elliptic curve, including scalar multiplication on the elliptic curve, encoding phase processing, and the mapping phase. To illustrate, MCS schemes in [53,54] and similar proposed schemes in [55,56] use ECC without elaborating on the processing associated with each phase. For this reason, these schemes provide reduction on the processing computation and power consumption. However, many proposed schemes have enhanced the phases involved in ECC. For instance, refs. [57,58] introduced efficient algorithms to increase scalar multiplication performance on the elliptic curve. Equally important, other proposed schemes have described how the ECC phases can be performed, but they neglect to use (or to elaborate on) the approach to padding bits used to secure the mapping phase [59,60]. Moreover, many studies have provided details on the mapping phase, specifically the padding bits step, where the mapped point requires additional bits to secure the ciphertext mapped to the elliptic curve. The remainder of this review of related work focuses on these studies and, in particular, addresses the process of how the padding bits step can improve the transmission performance and reduce the size of the ciphertext.

Four decades ago, ECC was proposed by Koblitz and Miller [61], and it began to be widely used in the beginning of this millennium [62,63,64,65,66,67,68]. The first curve used in ECC was introduced by Koblitz in 1987 [69]. Koblitz specified the steps needed to map plaintext to the suggested curve. More specifically, the author proposed several methods to ensure the successful mapping of $x_{1}$ to the elliptic curve. Some of these methods necessitate a large computational overhead, which means they are unsuitable for environments that rely on low computing power devices. However, Koblitz proposed one method that was suitable for such environments, where $x_{1}$ is assumed to be an integer value. Thus, in this method, it is concatenated with 3 digits. In turn, the new $x_{1}$ value is safely incremented until $y_{1}$ is obtained. Once $\left(x_{1},y_{1}\right)$ is mapped to the elliptic curve, the $x_{1}$ is straightforwardly decoded by removing the concatenated three digits. Resultantly, concatenating three digits is equivalent to padding 24 bits to $x_{1}$, which corresponds to $2^{24}$ rounds to find the corresponding $y_{1}$.

In 2018, Tiwari & Kim [70] introduced a novel approach using DNA-based ECC. In this approach, genome sequences are used to assign different values to each character set in the message. In turn, every *m* is mapped to the elliptic curve by multiplying it with a random integer *r* such that mr<p, where *p* is a large prime. Furthermore, if the mapping of mr fails, then the value is incremented by 1 and the mapping method is repeated. Moreover, when the mapping process is completed, the authors described the reverse approach to represent the original value of *m* by taking the ceiling value after dividing the mapped point by *r*. Using this approach, *m* can be secured when mapping it to the elliptic curve for *r* rounds. However, a limitation of this approach is that the authors failed to specify requirements regarding the acceptable size of *r*. Similarly, the authors did not describe the impact of selecting an improper value of *r*.

Message mapping and reverse mapping in ECC was introduced by Sengupta & Ray in 2016 [71]. In their paper, the authors gathered the characters taken from a message into a group to map it to the elliptic curve. Subsequently, the authors suggested concatenating *N* bits to this group of characters. The authors stated that the value of *N* enables the counting of the number of rounds needed to map the group, where the number of rounds amounts to $2^{N}$. In addition, the authors stated that no known algorithm existed for finding the optimal value of *N*, and the only approach involved determining the coordinates on the curve (i.e., solving the ECDLP). Thus, the authors used an 8-bit value of *N* as they suggested that the maximum value of *N* is always less than a certain value. They observed that 8 bits were adequate for use as the maximum of that value. Using this value, mapping to the EC gives $2^{8}$ rounds for guaranteeing successful mapping to the elliptic curve.

In 2009, King [72] described an approach for mapping a message to an elliptic curve using a probabilistic strategy. The author used the binary representation of a message *M* in the probabilistic equation as the value of $x_{i}$. Thus, to find the corresponding $y_{i}$, the approach involves computing $x_{i}^{3}+ax_{i}+b$. If $y_{i}$ has a square root, then the $x_{i}$ has mapped to EC and it has a corresponding $y_{i}$. However, If $y_{i}$ mapped in the first round, then the mapping method continues to compute $y_{i}$ by incrementing $x_{i}$ until $y_{i}$ is found. The number of rounds is defined by a random integer *k*, which is used in $x_{i}=M\times k$. To derive the original value of $x_{i}$, the floor value of $\frac{x_{i}}{k}$ is needed. In certain cases, for a given *k*, it is not possible to map $y_{i}$ to the elliptic curve because *k* is too small. Similarly, an overly large value of *k* increases of size overhead of $x_{i}$, resulting in an increase in the data transmission overhead. It is important to note that the author defined the probability of $y_{i}$ being mapped successfully based on $\frac{1}{2^{k}}$.

## 3. The Proposed Scheme-Mapping and Padding Method to ECC

The proposed scheme consists of nine phases started by generating parameters; then encoding messages to numerical values, followed by mapping these values to EC, the encrypting phase, signing the encrypted message. The rest of the phases are the reverse operations of the previous phases, beginning with verifying the signature of the received cipher text, then the decryption phase, followed by decoding phase, and converting the received points into plaintext. The main goal of this research is to study the padding methods in mapping phase where many proposed schemes did not provide a comprehensive details about the used padding method. In addition, it is noteworthy that the padding bits size is an important factor on mapping phase, where many of current studies neglect to provide it more focus and as a result it may lead to increase the size of padding bits which increase the size of mapped points or decrease the size of padding bits which result to increase the probability mapping phase failure.

Padding phase is an important phase in the ECC to make sure that the mapping points to EC are successfully restored in the decoded phase. To map a character to the EC, first it needs to convert to numerical value to map it to EC. Afterward, the converted value needs the padding phase to make sure that, if the first mapping operation to the EC failed, it is safe to increase it by one to try the mapping operation again. Finally, the mapped point can restored easily to the original value in the decoded phase. Padding phase can affect the performance of ECC schemes implemented on devices in constrained environments in two ways. The first way results from the size of padding bits needed to guarantee that the encoded points map successfully to the elliptic curve. The size of the padding bits represents the number of rounds that the encoded points can increment without affecting the original value. The second way relates to the fact that the padding bits can affect the ECC scheme in terms of the process by which the encoded points are padded. Two methods are commonly employed to pad encoded points, namely the probability method and concatenating method. The first method pads bits by multiplying the encoded points by a random integer *k*, where the results can increment *k* times to map the encoded points to the elliptic curve safely. The original value is retrieved by taking the floor value after dividing the mapped points by *k*. The second method involves directly concatenating *n* bits to the encoded points. In this approach, $2^{n}$ rounds can be safely incremented. After mapping the encoded points, the original value is retrieved by removing the *n* concatenated bits.

### 3.1. Generating System Parameters

The main task of this phase is to generate the parameters and constructs the encryption key between the two parties. Table 1 describes the symbols used in the proposed scheme.

**Figure 2:**
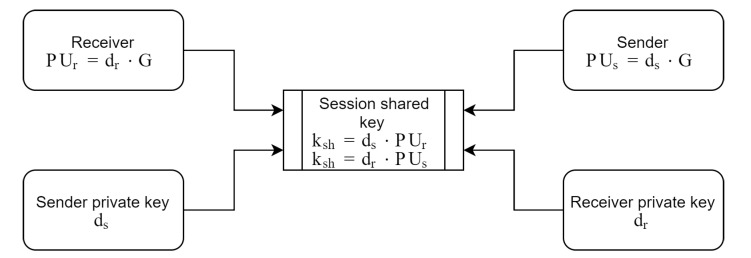
Generate the shared key between two parties.

The key $k_{sh}$ that shared between two parties used to encrypt mapped points on the elliptic curve. In order to create this key, the sender uses his/her private key $d_{s}$, and multiply it to recipient’s public key $PU_{r}$, thus, the sender generates $k_{sh}$. Benefiting from ECDLP, the recipient generates the $k_{sh}$ in the same way by multiplying his/her private key $d_{r}$ to sender’s public key $PU_{s}$. This process illustrated in Figure 2.

### 3.2. Encoding and Mapping the Message to EC

The EC encoding and mapping approaches steps are enhanced to increase the performance and decrease the computation efforts. Each plaintext is divided into set of blocks notated by *B*, and each block *B* has *N* characters. The calculation of *N* is the floor of the size of prime number generated from Table 1 subtracted by 8 divided by 8. The following equation describes the process:

(2) $$N\leq \lfloor \frac{p-8}{8}\rfloor$$

Similarly, the count of blocks *B* needed is the division of the total number of characters in the message *M* by the size of characters for each block *N*. The following equation describes the process:

(3) $$B=\lceil \frac{M}{N}\rceil$$

The encoding step for each block *B* is completed by converting its ASCII code to binary to perform cipher block chaining (CBC) to secure the cipher texts against several encryption attacks. Equally importantly, the mapping phase needs to append set of bits to each encoded blocks. This step is an important process to secure and guarantee the successful mapping to EC. The details of padding bits is described in subsection D.

### 3.3. Authenticated Encryption of the Mapped Points

Many proposed schemes consider the mapping phase is appropriate and enough to secure the transmitted message. However, mapping points to an EC is the first step to secure the cipher text and it is needed for the encryption step which is the addition of encryption key with the mapped points as follows: $C_{M}=k_{sh}+Mapped\;points$. Following that, it is necessary to maintain the integrity of the cipher text $C_{M}$ using the ECDSA. The first step is to obtain $e=HASH(C_{M})$ and take the left most p bits of *e*. Then, the second step is randomly select *k* and calculate (x,y)=kG, then calculate *r*, where r=xmodp and r≠0. Finally, the signed cipher text is the pair of (r,s) where $s\;=\;\left(z+d_{s}\;\times \;r\right)\;k^{-1}$.

### 3.4. Mapping Phase and Padding Encoded Points

The hardiness of ECDLP resides in the mapping phase and the correctness of the steps of mapping points to EC. Failing of mapping point to EC means that the encrypted data using ECC is weak. Several security issues are raised by failing to map points to EC, for instance, ignore the padding bits that result in failing to map points to EC by 50%. Equally importantly, pad encoded points with a small size of bits raise the percentage of the fail of the mapping phase. Similarly, the increase of the size of padding bits leads to an increase in the computation and transition overhead, particularly for low computation devices (such IoT) that need to deal with a huge amount of data (such as big data processing).

In the review of related work, one of two padding methods was used in all of the proposed schemes: the probability method, which relies on integer multiplication, and the concatenation method, which combines the encoded points with a specific number of padding bits. Hence, it is worth evaluating the performance of both methods to identify viable ways in which minimise the computational overhead, particularly for devices operating in constrained environments. This performance evaluation constitutes the focus of the next two subsections.

#### 3.4.1. Probability Method

**Figure 3:**
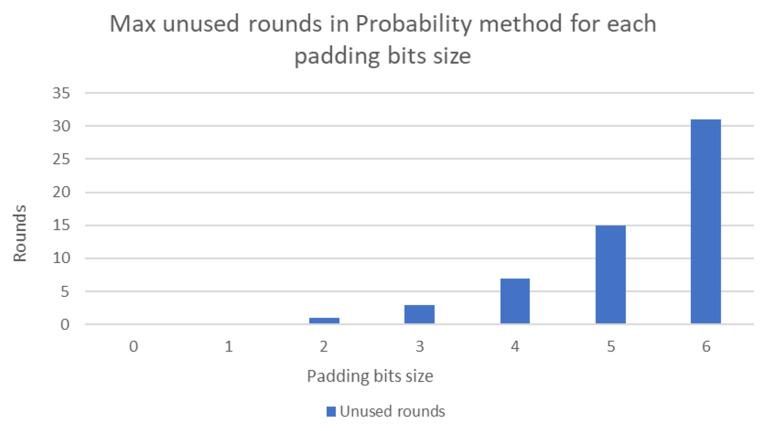
Maximum number of unused rounds for various padding bit sizes.

The main computational overhead associated with the probability method arises from the need to multiply the encoded points by a random integer *k*. The value of *k* represents the number of rounds needed to map the encoded points. For instance, if the number of rounds needed to map the encoded points is 25, then k=25. However, the value of 25 increases the number of padding bits that must be added to the encoded points by ⌈log25⌉=5. Moreover, padding with 5 bits provides $2^{5}=32$ rounds. Resultantly, 32−25=7 rounds remain unused when multiplying the encoded points by 25. Figure 3 illustrates the maximum number of unused rounds for padding bit sizes ranging from 0 to 6.

#### 3.4.2. Concatenation Method

**Figure 4:**
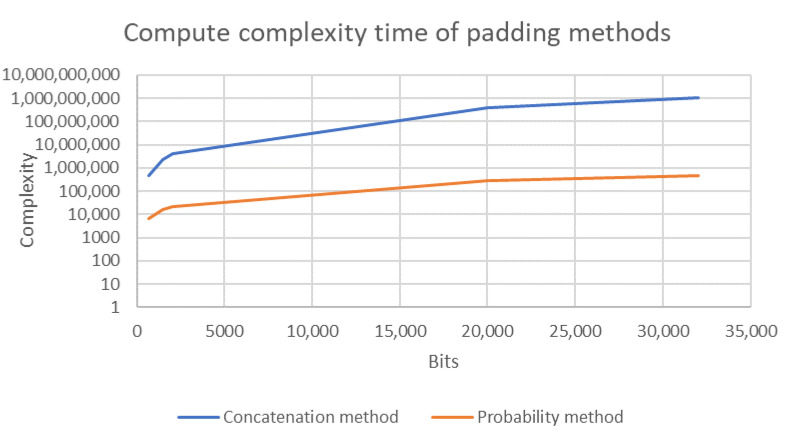
Complexity of probability and concatenation padding methods.

The main computational overhead associated with the concatenation method arises from the need to append padding bits to the encoded points. In this method, the number of appended bits is selected based on the number of rounds needed to secure the mapping phase of the encoded points. The appended bits of size 5 is 00000, thereby providing up to $2^{5}=32$ rounds to secure the padding phase. In the concatenation phase, no rounds are unused because every appended bit is used. However, the concatenation method is an expensive approach, especially for low computing power devices. The complexity of string concatenation is computed as $O(n^{2})$ [73], where *n* is the number of padding bits. Contrastingly, the complexity of integer multiplication is computed as O(nlogn) [74], where *n* is the size of the multiplication value. Figure 4 compares the complexity of the two methods.

### 3.5. Our proposed Padding Method

**Figure 5:**
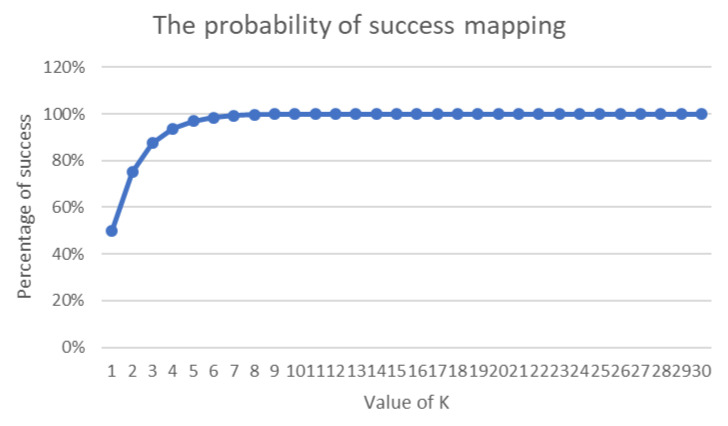
King’s [72] probability value of successful mapping.

Many ECC schemes have proposed several sizes for the padding bits that are used to secure the mapping phase to the elliptic curve. However, there is no comprehensive study of the suitable size of such bits. Certain schemes have proposed that 8 bits should be padded to each character mapped to the elliptic curve, while other schemes have proposed adding 8 padding bits to a set of aggregate characters. Similarly, schemes that use the probability method typically fail to specify the value of *k*, thereby leading either to an increase in the size of the transmitted data overhead or to an increase in the probability of a failed mapping. Based on King’s [72] research, the successful mapping occurs in the 8th round, as illustrated in Figure 5.

In this research, several experiments were conducted to evaluate the size of the padding bits required to increment $x_{i}$ safely and to find the corresponding $y_{i}$ value. Similarly, this research also aims to maximized the performance of the padding bits method and overcome the weakness of both methods mentioned in the previous sections. The experiment addressed the following cases:

- Case 1: Random 192-bit integer based on the requirements of secp192k1.
- Case 2: Random 224-bit integer based on the requirements of nistP224.
- Case 3: Random 256-bit integer based on the requirements of secp256k1.
- Case 4: Random 384-bit integer based on the requirements of secp384r1.
- Case 5: Random 512-bit integer based on the requirements of secp512r1.

For each case, the experiment was repeated 10 million times for each curve to evaluate the maximum number of rounds needed to successfully map the random number to the curve. The results show that, for certain random numbers, the maximum number of rounds was less than 25.

**Figure 6:**
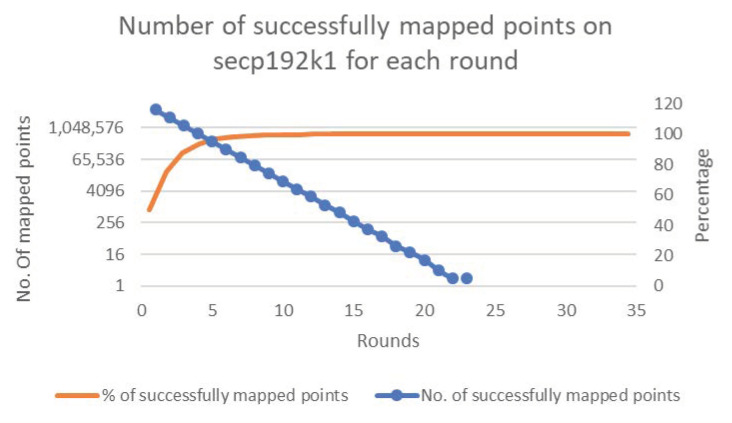
Case 1—Successfully mapped points count to secp192k1.

**Figure 7:**
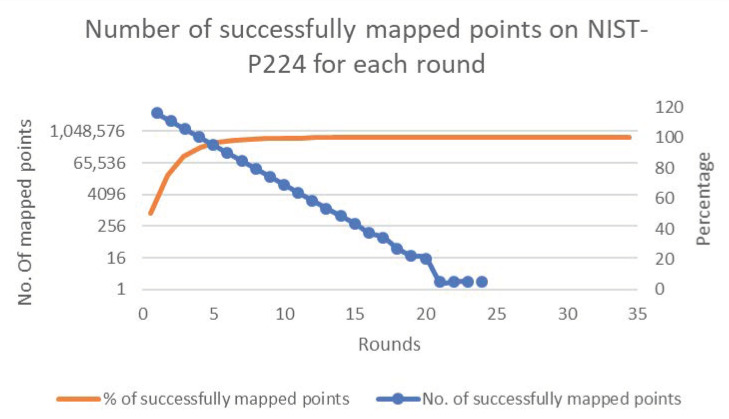
Case 2—Successfully mapped points count to nistP224.

**Figure 8:**
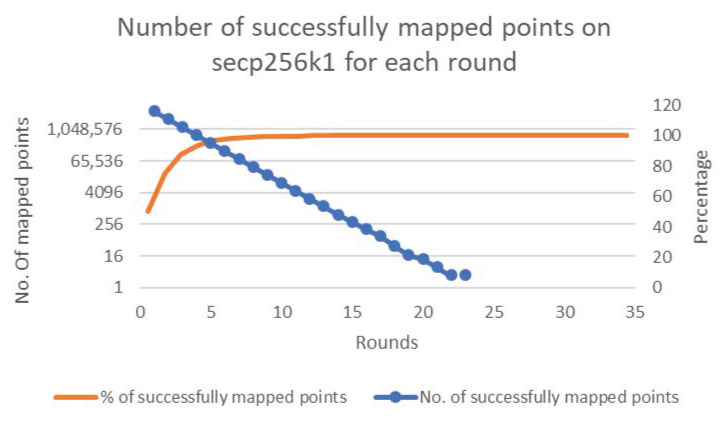
Case 3—Successfully mapped points count to secp256k1.

**Figure 9:**
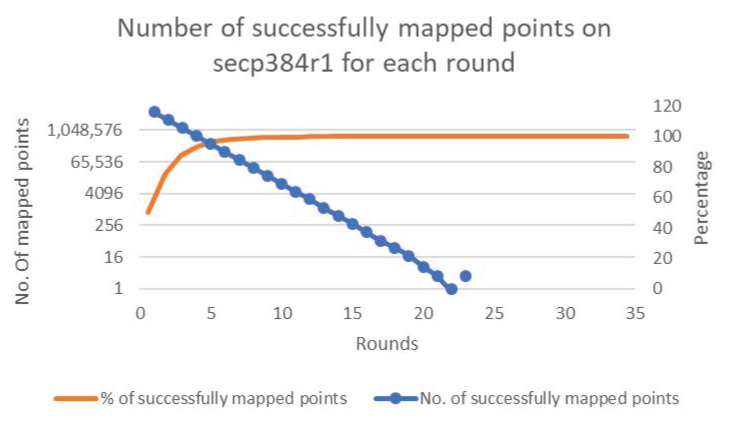
Case 4—Successfully mapped points count to secp384r1.

**Figure 10:**
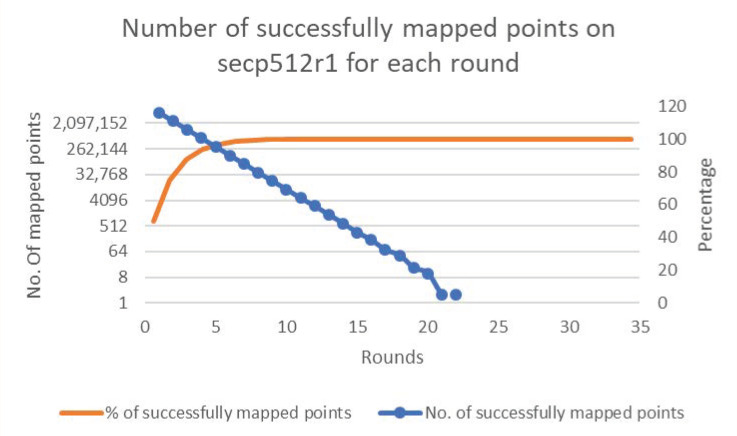
Case 5—Successfully mapped points count to secp512r1.

Figure 6, Figure 7, Figure 8, Figure 9 and Figure 10 present the results of the experiments for each of the cases. The figures show the percentages and numbers of successfully mapped points for each round (the orange curves and blue columns, respectively).

The experiments presented showed that, for every case, the number of rounds did not exceed 25. Thus, the size of padding bits that are needed to secure the mapping phase should not exceed $\left\lceil \log_{2}25\right\rceil =5$. Thus, it is possible to overcome the weakness in the probability method where the unused rounds may increase based on the value of *k*. In addition, there is a weakness in the concatenation method, which increases the computation overhead. This research provides the enhancement of the probability method by adding one step to choose $k=2^{\left\lceil \log k\right\rceil }$. The Algorithm 1 describes this process:
**Algorithm 1:** The proposed enhanced probability method algorithm.**Input**: MappingpointsM**Output**: Paddedmappingpointsx1 
Randomlyselectvaluek;
2 
$let\;k=2^{\left\lceil \log k\right\rceil }$;
3 
forj=0tok−1do;
4 
letx=M×K+jmodp;
5 
ifxmappedtoECthenbreak;
6 
ifj<kreturnxelsereturnfailed;


The enhanced probability method gains the strength of the concatenation and probability methods. In addition, it overcomes the weakness of both methods to increase the performance of the proposed system. What we mean by the strength of concatenation method is the number of rounds for the size of appended bits as depicted in Figure 3. Similarly, the strength of probability method means the redaction of the complexity that offered by concatenation method as depicted in Figure 4. In the following section, we provide the performance evaluation for the enhanced probability method. It is worth mentioning that, for computation evaluation, both the enhanced probability and probability methods provide the same result. However, the enhanced probability method overcomes the weakness of the probability method in terms of unused rounds in comparison with the size of appended bits.

## 4. Performance Evaluation

**Figure 11:**
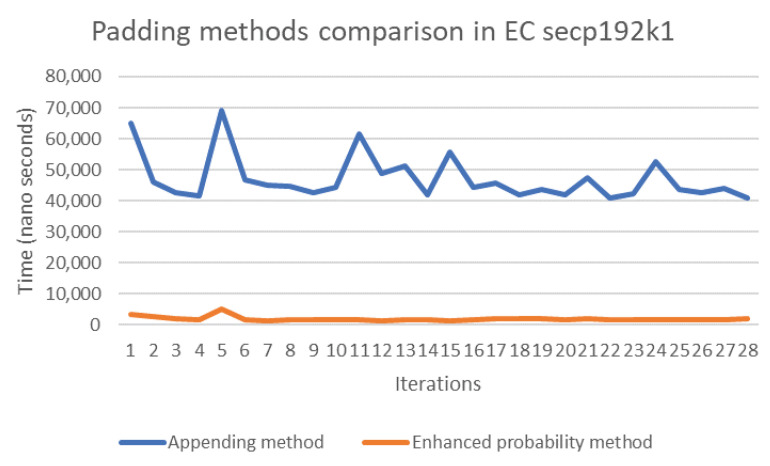
Performance evaluation for padding methods on secp192k1.

**Figure 12:**
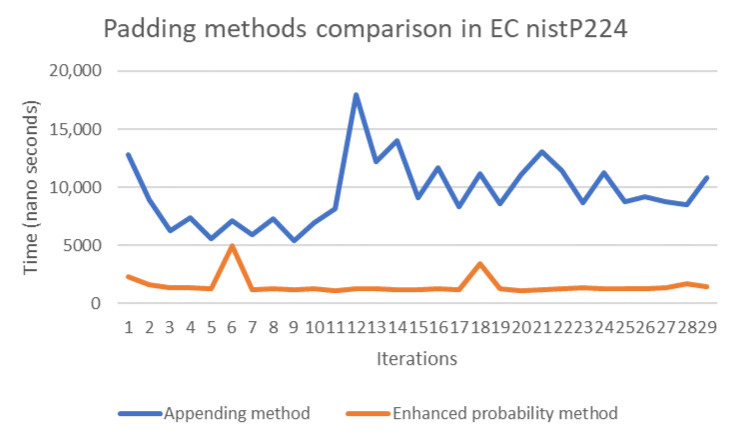
Performance evaluation for padding methods on nistP224.

**Figure 13:**
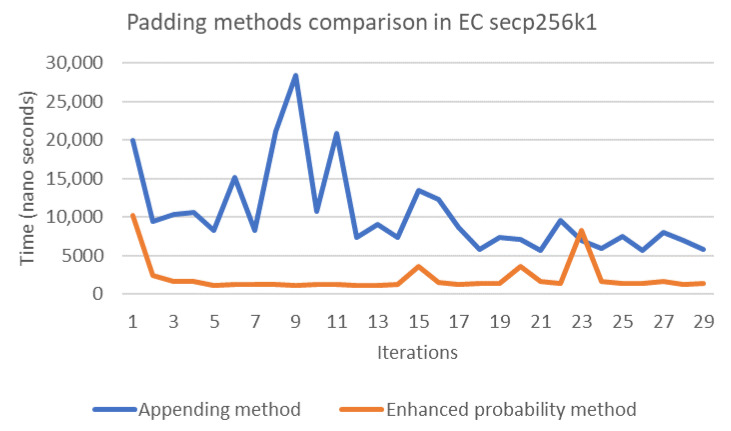
Performance evaluation for padding methods on secp256k1.

**Figure 14:**
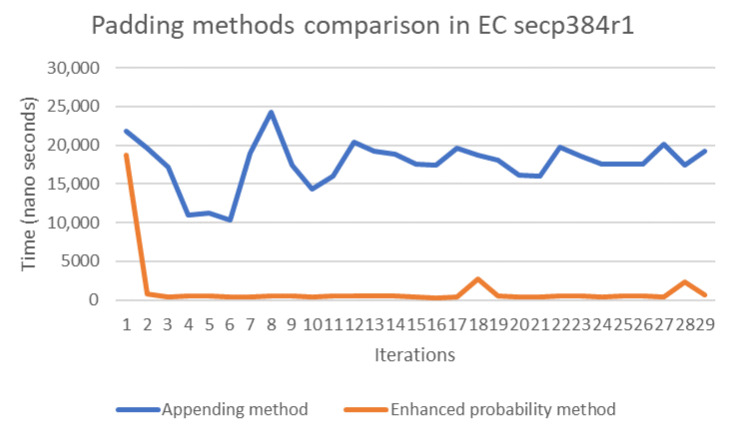
Performance evaluation for padding methods on secp3841.

**Figure 15:**
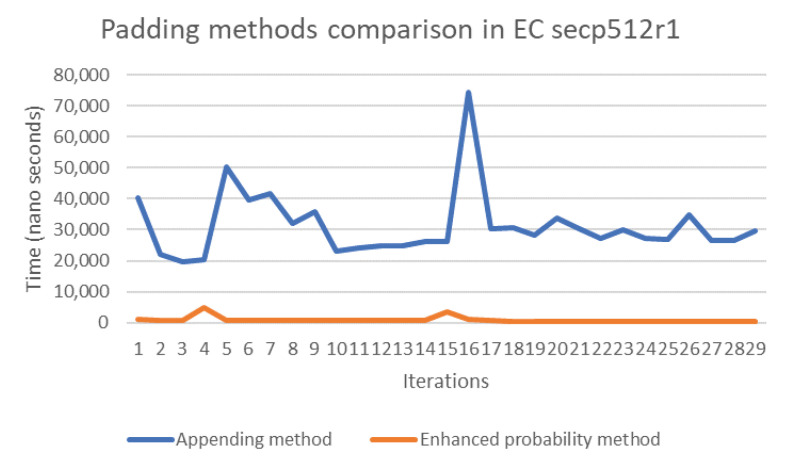
Performance evaluation for padding methods on secp512r1.

The probability and concatenation methods each have strengths and weaknesses in terms of their performance. Thus, in order to compare these methods, we simulated both approaches by writing Java code to compute the evaluation of both methods’ performance on several elliptic curves, namely secp192k1, nistP224, secp256k1, secp384r1, and secp512r1. Figure 11, Figure 12, Figure 13, Figure 14 and Figure 15 illustrate the results of this performance evaluation, indicating that, for all evaluated elliptic curves, the concatenation method (depicted in blue) required greater computational effort when compared to the enhanced probability method. Specifically, the computational requirement for the concatenation method was 35 times greater compared to the enhanced probability method for secp192k1, and it was 10–20 times greater for the other elliptic curves. The probability methods provide the same results as the enhanced probability method in terms of the computation evaluation test.

**Figure 16:**
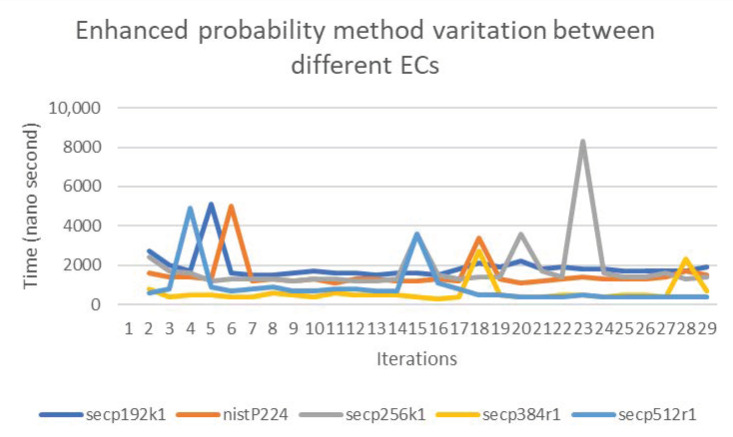
The variation between the set of Elliptic Curves (ECs) in the enhanced probability method.

**Figure 17:**
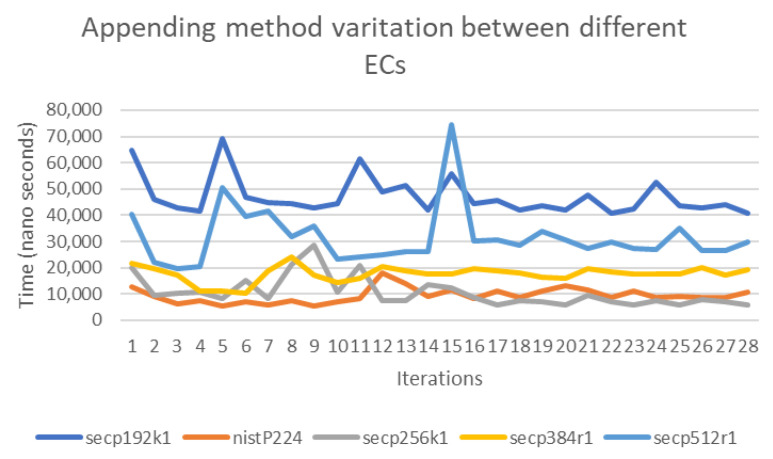
The variation between the set of ECs in the concatenation method.

The previous findings motivated a direct comparison of the computational overhead associated with each of the elliptic curves for every padding method. For instance, we were interested in quantifying the increase in computational requirements based on the elliptic curve used. Figure 16 shows the variation between the computation on all elliptic curves for the enhanced probability method, indicating differences of less than 3 times between the highest and lowest loads. Similarly, Figure 17 shows the variation between the computation on all elliptic curves for the concatenation method. As the figures indicate, the differences increased in comparison with the enhanced probability method, and the variation between the highest and lowest loads reached 8 times.

**Figure 18:**
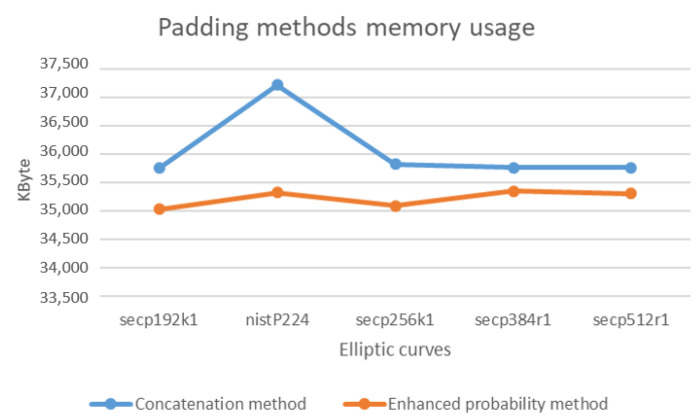
The Memory usage on padding methods using the set of ECs.

Similarly, a comparison between Enhanced probability method and Concatenation method on term of Memory usage. As in the previous evaluation test, we measure the memory space utilized for using both methods on the same set of EC. As a result, the enhanced probability method uses less memory space than concatenation method. This result is depicted in Figure 18.

**Figure 19:**
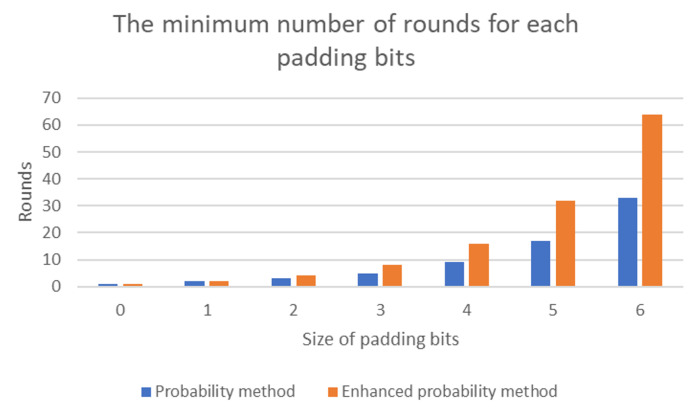
Number of minimum rounds for each size of appended bits.

In the other hand, the evaluation of the enhanced probability method in terms of minimum rounds to the size of appended bits is depicted in Figure 19. In this comparison, the enhanced probability method provides more rounds than the probability for each padding bit. For instance, for 5 bits appended to encoded points, the enhanced probability offers minimum 32 rounds. However, for the same same size, the probability method offers minimum 17 rounds. Thus, the enhanced probability method provides a better chance to map encoding points to EC than the the probability method.

To illustrate the overall comparison of the mapping methods, we summarize our findings in Table 2. In this table we show the detailed performance evaluation between our proposed method, (the enhanced probability method), the probability method and the concatenation method. It is clear that our proposed method is better than the other methods by gaining the strength features of those methods.

It is worth mentioning that, in our experiments, we used the Bluej Java Development Environment to code the mapping and padding phase. The environment used to test the code is based on Windows 10, and, in terms of hardware, Intel Core i7-4510U, 128GB SSD, and 8GB RAM.

## 5. Conclusions

This paper examined considerations relating to the optimal use of padding bits in the ECC mapping phase. It emphasised the importance of padding bits, particularly in terms of their performance implications. Additionally, the consequences arising from the improper addition of padding bits to encoded points were illustrated. Many proposed schemes choose a padding method without a clear explanation. Furthermore, most schemes tend not to provide details about the chosen sizes of padding bits. Therefore, this research sought to determine the optimal size of padding bits for the secure mapping of encoded points to an elliptic curve. It identified and enhanced a padding method that increases device performance and reduces the computational overhead without undermining security. Moreover, an evaluation of simulation performance was provided to illuminate and support the research findings.

In future work, the implications of implementing the suggested padding bits and enhanced mapping method in a real-world environment will be studied. The authors will also compare the computation results obtained in a real-world environment against those reported in this study’s simulation.

## Figures and Tables

**Table 1:** List of symbols used to generate scheme parameters.

Symbol	Description
$d_{s}$	Sender private key
$d_{r}$	Recipient private key
*G*	Base point on elliptic curve
$PU_{s}$	Sender public key = $d_{s}\times G$
$PU_{r}$	Recipient public key = $d_{r}\times G$
*p*	Large prime number (192-bit)
a,b	EC coefficients, s.t. $4a^{3}+27b^{2}\;mod\;p\neq 0$
*H*	Hash function to sign the message $C_{M}$
$k_{sh}$	Shared session key
*M*	Total number of characters in the message
*B*	Number of blocks for each message
*N*	Number of characters on each block
IV	Initial vector randomly selected (192-bit)
*k*	Randomly securely selected from [1,p−1]
$C_{M}$	The encrypted message

**Table 2:** The comparison between mapping methods.

Criteria	1	2	3
Number of rounds to value k	k	$2^{\left\lceil \log k\right\rceil }$	$2^{\left\lceil \log k\right\rceil }$
Provide max number of rounds	N	Y	Y
RAM usage per operation	Less	High	Less
Variation between different ECCs	Less	High	Less
CPU utilization per operation	Less	High	Less
Complexity of the method	O(nlogn)	$O(n^{2})$	O(nlogn)

1: Probability method, 2: Concatenation method and 3: our proposed method (Enhanced probability method)

## Abbreviations

The following abbreviations are used in this manuscript:

EC	Elliptic Curve
ECC	Elliptic Curve Cryptography
IoT	Internet of Things
IV	Initial Vector
MCS	Mobile Crowed-sourcing System
AE	Authenticated Encryption
ECIES	Elliptic Curve Integrated Encryption Scheme
ECDLP	Elliptic Curve Discrete Logarithm Problem
